# Osteoarthritis Affects Mammalian Oogenesis: Effects of Collagenase-Induced Osteoarthritis on Oocyte Cytoskeleton in a Mouse Model

**DOI:** 10.1155/2021/8428713

**Published:** 2021-11-09

**Authors:** Anton Ivanov Kolarov, Irina Valcheva Chakarova, Valentina Prodanova Hadzhinesheva, Venera Pantaleeva Nikolova, Stefka Metodieva Delimitreva, Maya Dyankova Markova, Ralitsa Stefanova Zhivkova

**Affiliations:** Department of Biology, Medical Faculty, Medical University—Sofia, 2 Zdrave Str, 1431 Sofia, Bulgaria

## Abstract

Known as a degenerative joint disorder of advanced age affecting predominantly females, osteoarthritis can develop in younger and actively working people because of activities involving loading and injuries of joints. Collagenase-induced osteoarthritis (CIOA) in a mouse model allowed us to investigate for the first time its effects on key cytoskeletal structures (meiotic spindles and actin distribution) of ovulated mouse oocytes. Their meiotic spindles, actin caps, and chromatin were analyzed by immunofluorescence. A total of 193 oocytes from mice with CIOA and 209 from control animals were obtained, almost all in metaphase I (M I) or metaphase II (MII). The maturation rate was lower in CIOA (26.42% M II) than in controls (55.50% M II). CIOA oocytes had significantly larger spindles (average 37 *μ*m versus 25 *μ*m in controls, *p* < 0.001), with a proportion of large spindles more than 64% in CIOA versus up to 15% in controls (*p* < 0.001). Meiotic spindles were wider in 68.35% M I and 54.90% M II of CIOA oocytes (mean 18.04 *μ*m M I and 17.34 *μ*m M II versus controls: 11.64 *μ*m M I and 12.64 *μ*m M II), and their poles were approximately two times broader (mean 6.9 *μ*m) in CIOA than in controls (3.6 *μ*m). CIOA oocytes often contained disoriented microtubules. Actin cap was visible in over 91% of controls and less than 20% of CIOA oocytes. Many CIOA oocytes without an actin cap had a nonpolarized thick peripheral actin ring (61.87% of M I and 52.94% of M II). Chromosome alignment was normal in more than 82% in both groups. In conclusion, CIOA affects the cytoskeleton of ovulated mouse oocytes—meiotic spindles are longer and wider, their poles are broader and with disorganized fibers, and the actin cap is replaced by a broad nonpolarized ring. Nevertheless, meiotic spindles were successfully formed in CIOA oocytes and, even when abnormal, allowed correct alignment of chromosomes.

## 1. Introduction

Osteoarthritis is an age-related degenerative condition of joints characterized by cartilage destruction, abnormal bone remodeling, pain, swelling, and limited range of motion. It affects a significant proportion of older people, has a negative impact on their quality of life and workability, and leads to increased medical costs and care dependency.

Osteoarthritis depends not only on age but also on sex, being more common in women than in men [1, 2]. Other known risk factors include obesity, number of pregnancies and births, and hormone replacement therapy. Studies on the combined action of risk factors have revealed additive and multiplicative effects when reproductive and hormonal factors of premenopausal female life (e.g., a high number of pregnancies and oral contraceptives usage) are followed by postmenopausal factors such as hormone replacement therapy and/or obesity [1, 3–5]. Birth parameters such as preterm delivery and low body weight of the newborn are associated with an increased risk of osteoarthritis in the adult life of the individual, presumably as a result of abnormal hip bone development [6]. These findings show that osteoarthritis should be regarded not as an isolated process of joint degeneration in advanced age but as a complex condition developing in the context of the whole organism under the influence of various factors over the lifetime.

Although osteoarthritis has been historically defined as a noninflammatory degenerative joint disease, recent data have shown an important role concerning inflammatory pathways and mechanisms. The progression of cartilage destruction and synovial membrane damage is associated with the development of both humoral and cell-mediated immunity: complement activation, proinflammatory cytokines, and infiltration by activated macrophages and T-cell subpopulations [7]. Some authors describe osteoarthritis as a low-grade local inflammatory process based on the presence in osteoarthritic joints of proinflammatory cytokines (a cohort of interleukins such as IL-1*β*, IL-6, IL-15, IL-17, IL-18, IL-21, tumor necrosis factor-alpha, and leukemia inhibitory factor) and chemokines that are not carried by systemic circulation [8], possibly including innate immunity mechanisms such as recognition of damage-associated molecular patterns and involvement of complement effectors that can mediate synovial macrophage and mast cell activation [9]. As immune factors of local significance, these inflammatory agents could be destructive and have been implicated in the chronic inflammation accompanying osteoarthritis [10]. Other researchers report that inflammatory changes in osteoarthritis are not locally restricted but generalize and become systemic. Markers of systemic inflammation such as elevated blood leukocytes [11] and adipokines [12] have been detected in patient sera, and comparisons of synovial and systemic inflammation markers suggest a positive correlation between serum and synovial fluid levels of C-reactive protein, IL-17, and blood leukocytes [13, 14].

Mice with collagenase-induced osteoarthritis (CIOA) are an adequate model of the human disease: they display the main osteoarthritic symptoms (articular cartilage degradation, subchondral bone sclerosis, osteophyte formation, and synovial inflammation and hyperplasia) similarly to affected humans, the development of the pathology is reproducible, and symptoms appear rapidly, which allows observations and studies within the short lifespan of the animals. Combined with the low cost and efficient breeding, these advantages make mice with collagenase-induced osteoarthritis a widely used and relevant animal model for studies on mechanisms of osteoarthritis development, the role of genetic predisposition, and potential treatment approaches [15, 16].

The possibility of osteoarthritis development in younger women engaged in joint-loading sports or arts raises the question about the potential impact of the condition on their reproductive ability. We have not found literature data related specifically to the effects of arthritis on oogenesis, but such effects are known to exist in other inflammatory disorders. Levels of anti-Müllerian hormone are lowered in women with systemic lupus erythematosus, indicating depletion of the ovarian reserve [17]. The MRL/MpJ (MRL/+) mice that spontaneously develop systemic autoimmunity have fewer follicles in their ovaries, compared to healthy animals [18]. In recent years, data are accumulating that the adverse effects of obesity and polycystic ovary syndrome on oogenesis are partly due to the low-grade chronic inflammation associated with these conditions [19].

In mammalian oogenesis, as in most other animals, microtubules organize the spindle in the absence of centrioles (which have degenerated). The microfilament (actin) cytoskeleton interacts with the meiotic spindle and mediates its migration to the periphery of the cell, leading to highly asymmetric cytokinesis and retention of most of the ooplasmic volume for the secondary oocyte. A peripheral accumulation of fibrillar actin (actin cap) anchors the spindle to the oocyte cortex. The ovulated oocyte is arrested at meiotic metaphase II, with the spindle beneath the actin cap and near the first polar body [20]. All these peculiarities require precise control of the ooplasm rearrangements and chromosome alignment for proper oocyte maturation and eventual fertilization.

The aim of the present study was to evaluate the quality and morphology of key cytoskeletal structures (meiotic spindles and actin cap) and chromosome alignment of ovulated oocytes from mice with collagenase-induced osteoarthritis in order to reveal the eventual effects of the pathological process on oogenesis.

## 2. Materials and Methods

Female outbred ICR, 8- to 10-week-old mice were used in the current investigation: 10 mice (the experimental, CIOA group) were anesthetized by sodium pentobarbital at a dose of 50 mg/kg intraperitoneally and injected by 2 IU/10 *μ*l collagenase (from *Clostridium histolyticum*, Sigma-Aldrich, Germany) into the tibiotalar intraarticular space to induce osteoarthritis (day 0). The inflammation and phases of arthritis (initial acute, developing inflammation, chronification, and established phase) were studied in earlier works, and our treated animals were used in their established phase of CIOA as previously described [14, 21]. Nine females (the control group) were not treated by collagenase; they were anesthetized and injected i.a. with PBS (sham) and then housed for 30 days at the same regimen as the CIOA group. Both CIOA and control groups of animals were kept according to the standard laboratory housing requirements. All experiments conformed to the recommendations of Bulgarian Food Safety Agency Guidelines no. 352 06.01.2012 and the international laws and policies of EEC Directive of 1986; 86/609/EEC and the recommendation 2007/526/EC. The protocols were approved by the Animal Care and Use Committee of the Institute of Microbiology, Sofia.

The two groups of female mice (controls and with CIOA) were subjected to hormonal ovarian stimulation to produce synchronously ovulated oocytes: intraperitoneal injection of follicle-stimulating hormone (Meriofert, IBSA Farmaceutici, Italy)—12 IU per animal at day 31 after CIOA— induction followed by injection of human chorionic gonadotropin (Choriomon, IBSA Farmaceutici, Italy)—14 IU per mouse, 48 h later. The hormonal doses were adjusted to the weight and age of animals. Mice were anesthetized and then sacrificed by cervical translocation 16–18 hours after the second injection (at day 34), and the ovaria and oviducts were collected.

Oviducts were manipulated on a hot plate (37°C) in Leibowitz medium (Sigma-Aldrich, Germany) supplemented with 0.3% bovine serum albumin (BSA, Sigma-Aldrich). Ampullae of the oviducts were punctured, and the ovulated eggs were harvested, washed in clean Leibowitz, and then treated with 0.5 mg/ml hyaluronidase (Sigma-Aldrich) in the same medium to remove the cumulus layers of corona radiata surrounding the oocytes. Corona-free oocytes were washed from the medium in washing buffer: phosphate-buffered saline (PBS), pH 7.2, with 0.3% BSA. Then, they were fixed in 2% paraformaldehyde (in PBS with 0.02% Triton X-100) for 45 min at 37°C. After being washed twice (10 min each), oocytes were stored overnight at 4°C, in 0.02% sodium azide supplemented washing buffer.

Both CIOA and control group-derived oocytes were subjected to indirect immunofluorescence to visualize their meiotic spindles, actin caps, and chromatin. They were processed as follows. A monoclonal mouse anti-*α*-tubulin antibody (clone DM1A, Sigma-Aldrich), diluted 1 : 1000 in diluting buffer (PBS, pH 7.2, with 0.3% BSA and 1% sodium azide), was applied for 45 min at 37°C. Then, incubation of tubulin with FITC-labelled anti-mouse IgG antibody (Sigma-Aldrich), diluted 1 : 200 in diluting buffer, was performed for 45 min at 37^o^C in a dark chamber, together with phalloidin-TRITC (Sigma-Aldrich, working concentration 1 *μ*g/ml) and Hoechst 33258 (Sigma-Aldrich, working concentration 8 *μ*g/ml) to visualize F-actin and chromatin of the same oocytes, respectively. The oocytes were then washed, gradually embedded in 5%, 10%, 30%, and 50% polyvinyl alcohol (Fluka, Germany), transferred to 100% polyvinyl alcohol on microscopic slides, and covered by coverslips. The epifluorescent reaction was analyzed by fluorescent microscopy (Axioskop 20, Zeiss, Göttingen, Germany). The meiotic stage of the oocytes (M II, M I, or other) as well as the status of meiotic spindle and fibrillar actin was registered. Selected oocytes were subjected to laser-scanning confocal microscopy (LSCM at 0.1 *μ*m optical sectioning, Leica TCS SPE, Leica, Germany). LAS AF (Leica Application Suite Advanced Fluorescence, Version 1.8.2) software was applied for precise measurements of key meiotic spindle parameters: their total length and width, as well as spindle pole width. Using the LSCM results as standards, we applied the same measurements to the epifluorescent images of oocyte spindles. The results were subjected to statistical analysis. Comparison of the categories of data between the groups was done with the Fisher–Freeman–Halton Exact Test of independence, and *p* values under 0.05 were considered statistically significant. Comparison of numerical data was done using independent samples *t*-test. Data analysis was performed using IBM SPSS Statistics for Windows, Version 27.0, IBM Corporation.

## 3. Results

A total number of 402 ovulated mouse oocytes were analyzed: 209 were obtained from the control animals and 193 from mice with CIOA. Their meiotic stage was estimated based on the fluorescent images. The proportion of metaphase I (M I) and metaphase II (M II) stages, the latter ratio being the maturation rate, differed between the groups: 41.15% M I (86 of 209) and 55.50% M II (116 of 209) in the controls versus 72.02% M I (139 of 193) and 26.42% M II (51 of 193) for the CIOA oocytes, *p* < 0.001 (Table 1). The percentage of earlier meiotic stages of the oocytes (germinal vesicle or germinal vesicle breakdown) was low in both groups (2.39% and 1.04%, respectively). Degenerated or unanalyzable oocyte structures were found in 0.96% of control and in 0.52% of the CIOA group. The metaphase oocytes, 225 in M I (86 controls and 139 CIOA) and 167 in M II (116 controls and 51 CIOA), were subjected to analysis of their meiotic spindle, chromatin alignment, and actin cap.

### 3.1. Meiotic Spindle Size and Poles

The spindle parameters were classified into categories based on the mean ±1 SD of the control oocytes. All mean ±1SD spindle parameters were classified as “normal.” The spindles longer than the mean length +1 SD of control oocytes were counted as “long” (i.e., longer than 33.78 *μ*m for M I and 32.18 *μ*m for M II), and all of the spindles shorter than the mean –1SD of controls were “short” (i.e., shorter than 18.74 *μ*m for M I and 17.25 *μ*m for M II). The categories of spindle and pole width were set as follows: spindles wider than the mean width +1 SD of controls were counted as “wide” (i.e., 14.16 *μ*m for M I and 15.32 *μ*m for M II) and spindles with width below the mean –1 SD of controls were counted as “slender” (i.e., 9.13 *μ*m for M I and 9.96 *μ*m for M II). Spindle poles wider than the mean pole width +1 SD (i.e., 5.50 *μ*m for M I and 5.53 *μ*m for M II) were classified as “broad,” and those with pole width below mean –1 SD (i.e., 1.70 *μ*m for M I and 1.59 *μ*m for M II) as “narrow.”

**Figure 1:**
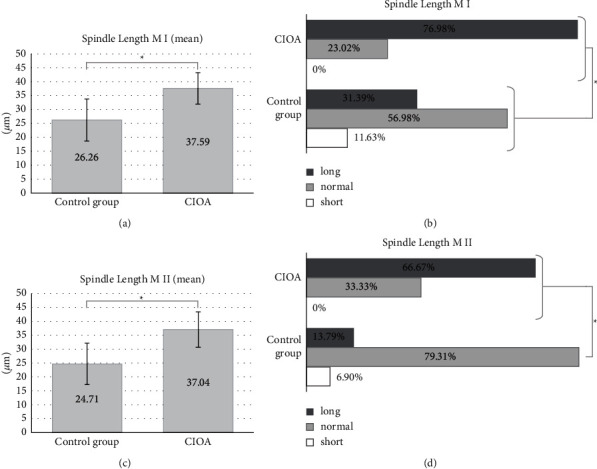
Meiotic spindle length. (a, b) Data for M I and (c, d) data for M II. (a) M I mean spindle length (total mean ± 1 SD), comparison of controls (26.26 ± 7.52 *μ*m, *n* = 86) and CIOA (37.59 ± 5.63 *μ*m, *n* = 139); independent samples *t*-test, ^*∗*^*p* < 0.001. (b) Distribution of M I spindle length categories in %. “Normal” range (mean ± 1 SD) from the control group. Control group (*n* = 86): short (*n* = 13), normal (26.26 ± 7.52 *μ*m, *n* = 60), long (*n* = 13); CIOA (*n* = 139): normal (26.26 ± 7.52 *μ*m, *n* = 50), long (*n* = 89); Fisher–Freeman–Halton Exact Test, ^*∗*^*p* < 0.001. (c) M II mean spindle length (total mean ± 1 SD), comparison of controls (24.72 ± 7.46 *μ*m, *n* = 116) and CIOA (37.04 ± 6.38 *μ*m, *n* = 51); independent samples *t*-test, ^*∗*^*p* < 0.001. (d) Distribution of M II spindle length category in %. “Normal” range (mean ± 1 SD) from the control group. Control group (*n* = 116): short (*n* = 8) < normal (24.72 ± 7.46 *μ*m, *n* = 94) < long (*n* = 14); CIOA (*n* = 51): normal (24.72 ± 7.46 *μ*m, *n* = 18) < long (*n* = 33); Fisher–Freeman–Halton Exact Test, ^*∗*^*p* < 0.001.

**Figure 2:**
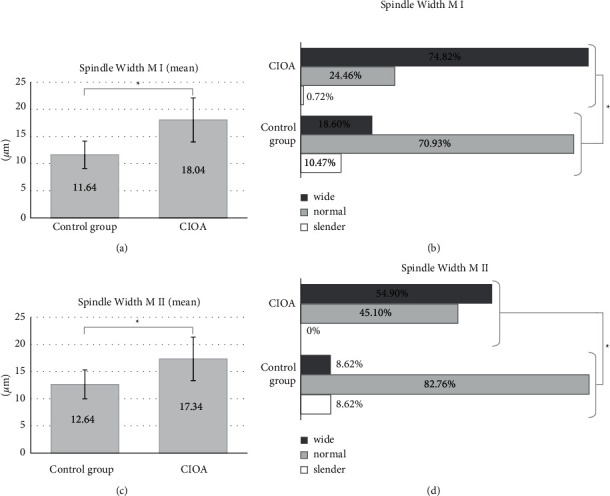
Meiotic spindle width. (a, b) Data for M I and (c, d) data for M II. (a) M I mean spindle width (total mean ± 1 SD), comparison of controls (11.64 ± 2.51 *μ*m, *n* = 86) and CIOA (18.04 ± 4.05 *μ*m, *n* = 116); independent samples *t*-test, ^*∗*^*p* < 0.001. (b) Distribution of M I spindle width category frequencies in %. “Normal” range (mean ± 1 SD) from the control group. Control group (*n* = 86): slender (*n* = 9), normal (11.64 ± 2.51 *μ*m, *n* = 67), and wide (*n* = 10); CIOA (*n* = 139): slender (*n* = 1), normal (11.64 ± 2.51 *μ*m, *n* = 43), and wide (*n* = 95); Fisher–Freeman–Halton Exact Test, ^*∗*^*p* < 0.001. (c) M II mean spindle width (total mean ± 1 SD), comparison of controls (12.64 ± 2.68 *μ*m, *n* = 116), and CIOA (17.34 ± 3.99 *μ*m, *n* = 51); independent samples *t*-test, ^*∗*^*p* < 0.001. (d) Distribution of M II spindle width category frequencies in %. “Normal” range (mean ± 1 SD) from the control group. Control group (*n* = 116): slender (*n* = 10), normal (12.64 ± 2.68 *μ*m, *n* = 97), and wide (*n* = 9); CIOA (*n* = 51): normal (12.64 ± 2.68 *μ*m, *n* = 23) and wide (*n* = 28); Fisher–Freeman–Halton Exact Test, ^*∗*^*p* < 0.001.

Compared to the controls, CIOA oocytes showed larger spindles illustrated by all analyzed M I and M II spindle parameters—the spindle length and width in the CIOA group differed significantly from those in controls (data concerning M I and M II spindle length are shown in Figure 1 and the width of spindles in Figure 2). The mean spindle length in the CIOA group (37.59 *μ*m ± 5.63 *μ*m for M I and 37.04 *μ*m ± 6.38 *μ*m for M II) was significantly larger than the mean length in the control group (26.26 *μ*m ± 7.52 *μ*m for M I and 24.71 *μ*m ± 7.46 *μ*m for M II spindles, data are shown in Figures 1(a) and 1(c), *p* < 0.001). Long spindles were significantly more prevalent among the CIOA cells (89 of 139 M I, 64.03% and 33 of 51 M II, 64.71%) than among the control oocytes (13 of 86 M I, 15.12% and 14 of 116 M II, 12.07%), *p* < 0.001. The normal spindle length, respectively, was more predominant in the controls—60 of 86 M I (69.77%) and 94 of 116 M II (81.03%), *p* < 0.001. Short spindles were absent in both M I and M II oocytes from the CIOA group (data shown in Figures 1(b) and 1(d)), *p* < 0.001.

Results concerning spindle width corresponded to the data for spindle length—the spindles of CIOA oocytes were wider than in controls and the mean values were significantly different between the two groups for both M I and M II. The mean spindle width for CIOA was 18.04 ± 4.05 *μ*m for M I and 17.34 ± 3.99 *μ*m for M II (Figures 2(a) and 2(c)). This significantly exceeded the width of the controls (11.64 ± 2.51 *μ*m for M I and 12.64 ± 2.68 *μ*m for M II), *p* < 0.001. Wide spindles were significantly more prevalent among CIOA cells (95 of 139, 68.35% for M I and 28 of 51, 54.90% for M II) than those among the control oocytes (10 of 86 M I, 11.63%, and 9 of 116 M II, 7.76%), *p* < 0.001. The normal spindle width predominated in controls (67 of 86 M I, 77.91% and 97 of 116 M II, 83.62%, *p* < 0.001), and the slender control spindles were relatively few (9 of 86 M I, 10.47%, and 10 of 116 M II, 8.62%) but still different from the slender spindles in CIOA oocytes—they were less than 1% in M I and absent in M II of CIOA group (Figures 2(b) and 2(d)), *p* < 0.001.

**Figure 3:**
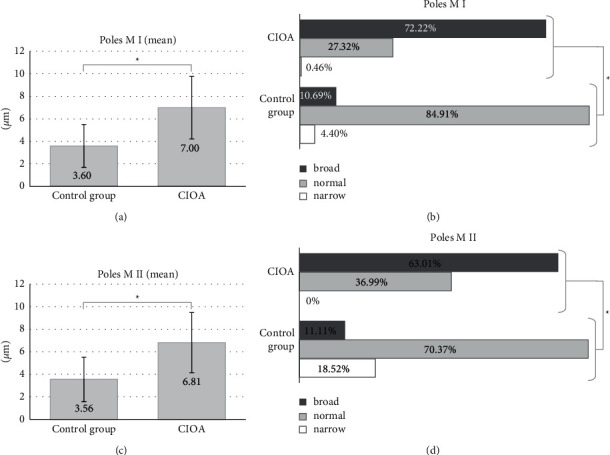
Spindle poles width. (a, b) data for M I and (c, d) data for M II. (a) M I mean pole width of the spindles. Spindle poles width (total mean ± 1 SD) of the control group (3.60 ± 1.90 *μ*m, *n* = 172) and CIOA (7.00 ± 2.78 *μ*m, *n* = 278); independent samples *t*-test, ^*∗*^*p* < 0.001. (b) Distribution of M I pole width category frequencies in %. “Normal” range (mean ± 1 SD) from the control group. Control group (*n* = 172): narrow (*n* = 19), normal (3.60 ± 1.90 *μ*m, *n* = 135), and broad (*n* = 18); CIOA (*n* = 278): narrow (*n* = 2), normal (3.60 ± 1.90 *μ*m, *n* = 75), and broad (*n* = 201); Fisher–Freeman–Halton Exact Test, ^*∗*^*p* < 0.001. (c) M II mean pole width of the spindles. Spindle poles width (total mean ± 1 SD) of the control group (3.56 ± 1.97 *μ*m, *n* = 232) and CIOA (6.81 ± 2.67 *μ*m, *n* = 102); independent samples *t*-test, ^*∗*^*p* < 0.001. (d) Distribution of M II pole width category frequencies in %. “Normal” range (mean ± 1 SD) from the control group. Control group (*n* = 232): narrow (*n* = 42), normal (3.56 ± 1.97 *μ*m, *n* = 150), and broad (*n* = 40); CIOA (*n* = 102): normal (3.56 ± 1.97 *μ*m, *n* = 38) and broad (*n* = 64). Fisher–Freeman–Halton Exact Test, ^*∗*^*p* < 0.001.

The spindle pole data are presented in Figure 3. The mean spindle pole width showed a clear and significant difference (Figures 3(a) and 3(c))—the poles in CIOA were approximately two times broader in all analyzed spindles (7.00 ± 2.78 *μ*m for CIOA M I versus 3.60 ± 1.90 *μ*m for control M I and 6.81 ± 2.67 *μ*m for COIA M II and 3.56 ± 1.97 *μ*m for control M II), *p* < 0.001. The broad spindle poles were about six times more prevalent for M I and about three times more prevalent for M II than the same stages in the control group (Figures 3(b) and 3(d)): 72.30% of CIOA M I (201 of 278) and 62.75% of CIOA M II (64 of 102) poles were broad compared to the proportion of broad poles of M I and M II spindles in the control group (18 of 172, 10.47% and 40 of 232, 17.24%, respectively), *p* < 0.001.

### 3.2. Spindle Morphology Peculiarities

**Figure 4:**
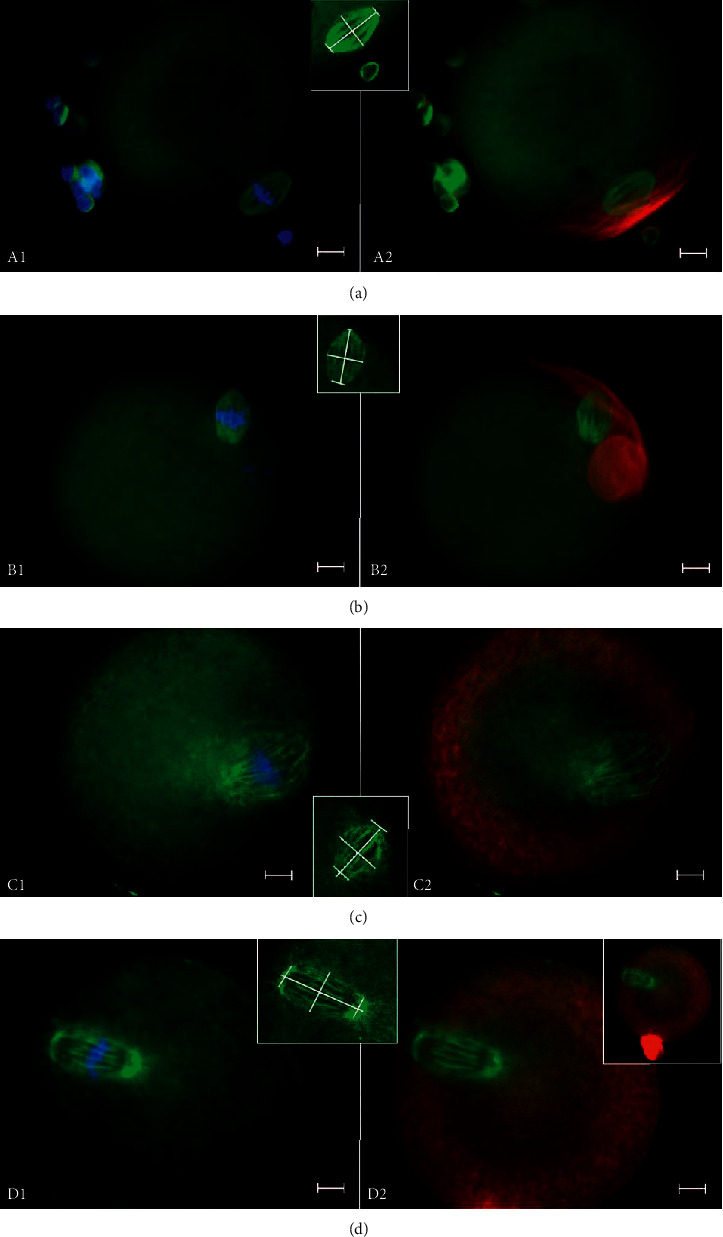
Fluorescent imaging of meiotic spindles (FITC-labelled tubulin), chromatin (stained by Hoechst 3325), and actin structures (Phalloidin-TRITC) in control and CIOA oocytes. Original magnification 1000x. Bar = 10 *μ*m. (a) An M I control oocyte with a normal spindle (green): A1, combined image with the aligned chromosomes (blue); A2, with the actin cap (red). A1 and A2 are epifluorescent images. The inset is an LSCM image of the same spindle used for measurement (25 *μ*m long and 15 *μ*m wide, pole widths 5 *μ*m and 4 *μ*m). (b) An M II control oocyte: B1, epifluorescence of a normal M II meiotic spindle in green, with the aligned chromosomes (blue); B2, the spindle and its actin cap adjacent (red), the polar body is seen too, by epifluorescence. The inset shows an LSCM image of the same spindle used for measurement (23 *μ*m long and 14 *μ*m wide, pole widths 3 *μ*m and 6 *μ*m). (c) An M I CIOA oocyte: C1, the spindle (green) is large (long and wide) with broad poles and disorganized microtubules around the spindle and its poles, and chromosomes (blue) are well aligned, by epifluorescence; C2, the same spindle combined with actin: a large ring (red) is seen instead of actin cap, by epifluorescence. Spindle size calculated using LSCM data: 45 *μ*m long and 31 *μ*m wide; poles: 14 *μ*m and 12 *μ*m. The inset shows a different M I CIOA spindle of normal length but wide shape and broad poles, with disorganized fibers. LSCM image measurements: 30 *μ*m long and 19 *μ*m wide; poles: 9 *μ*m each. (d) An M II CIOA oocyte. D1, the spindle (green) is large (long and wide), its poles are broad and with disorganized microtubules, and the chromosomes (blue) are well aligned, by epifluorescence. D2, the same spindle with an actin ring (red) instead of the cap (the additional right inset shows the whole oocyte with its polar body at original magnification 400x), by epifluorescence. The LSCM image inset shows the measurements of the spindle (39 *μ*m long and 21 *μ*m wide; poles: 10 *μ*m and 10 *μ*m).

Meiotic spindles of CIOA oocytes displayed a specific morphological feature (in addition to their enlarged size and wide poles, described above): part of their microtubules was disoriented and extended around the spindle and/or around the poles. This made these spindles look fuzzy, as shown in Figure 4. The proportion of these spindles was remarkable for the CIOA oocytes and significantly higher in this group than in controls: disoriented fibers around the spindle and/or around its poles were seen in nearly half in CIOA M I spindles (69 of 139, 49.64%) and more than half of CIOA M II spindles (30 of 51, 58.82%) compared to single or several disoriented fibers in 13.95% of control M I spindles (12 of 86) and 37.93% (44 of 116) of control M II spindles (Fisher-Freeman-Halton Exact Test, *p* < 0.001).

### 3.3. Actin Cytoskeleton

**Figure 5:**
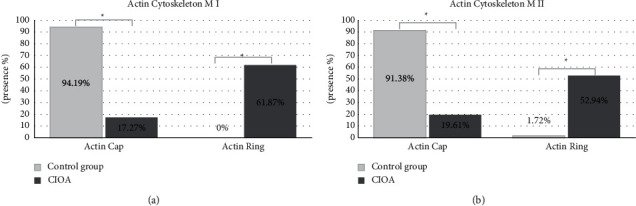
Actin cytoskeleton peculiarities. (a) Distribution of M I actin cap/ring, frequencies in %. Actin cap (grey) was present in 81 of 86 control oocytes and in 24 of 139 CIOA (black) oocytes. Actin rings were not present in any control oocytes and were present in 86 of 139 CIOA (black pattern) oocytes; Fisher–Freeman–Halton Exact Test, ^*∗*^*p* < 0.001. (b) Distribution of M II actin cap/ring, frequencies in %. Actin cap (grey) was present in 106 of 116 control oocytes and in 10 of 51 CIOA (black) oocytes. Actin ring was present in 2 of 116 control oocytes (grey pattern) and in 27 of 51 CIOA (black pattern) oocytes; Fisher–Freeman–Halton Exact Test, ^*∗*^*p* < 0.001.

Fibrillar actin distribution was analyzed by microscopic detection of the presence/absence of polarized cortical actin cap adjacent to the meiotic spindle. The data showed actin cap presence in 81 of 86 M I (94.19%) and 106 of 116 M II (91.38%) among the control group oocytes. CIOA group of oocytes had a significantly lower proportion of oocytes possessing an actin cap—24 of 139 M I (17.27%) and 10 of 51 M II (19.61%), *p* < 0.001. In many oocytes without an actin cap, a nonpolarized and relatively thick actin ring was observed in the oocyte periphery (Figure 4). This actin ring was seen mostly in CIOA oocytes: 61.87% of M I (86 of 139) and 52.94% of M II (27 of 51) had it. Actin rings were not seen in M I controls and only a low proportion of M II controls had it (2 of 116, 1.72%). Data concerning the presence of polarized actin cap and peripheral actin ring in CIOA and controls are shown in Figure 5. None of the oocytes possessing an actin cap had an actin ring and the opposite—oocytes displaying actin cytoskeleton as a thick peripheral ring showed no actin cap. Destroyed or undetectable actin cytoskeleton (no cap/ring) was registered in M I control (5.81%, 5 of 86), CIOA M I (21.58%, 30 of 139), M II control (6.90%, 8 of 116), and CIOA M II (27.45%, 14 of 51) oocyte groups.

### 3.4. Chromosome Alignment

The two groups of oocytes, the controls and CIOA, showed no significant difference in their chromosome alignment. A high proportion of good quality chromatin and properly aligned metaphase chromosomes as a plate at the equator of their meiotic spindles were seen: 84.88% M I (73 of 86) and 84.48% M II (98 of 116) among controls and 84.17% M I (117 of 139) and 82.35% M II (42 of 51) among CIOA. Misaligned chromosomes were registered in 11.63% (10 of 86) M I and 9.48% (11 of 116) M II for controls. CIOA oocytes had a slightly higher proportion: 14.39% M I and 13.73% M II with misaligned chromosomes. Oocytes with poor quality and degenerative appearance of their chromatin were a relatively small proportion and showed no significant difference between controls and CIOA.

## 4. Discussion

The present study provides, to our knowledge, the first data concerning the impact of osteoarthritis on mammalian oogenesis. Using a mouse model of collagenase-induced osteoarthritis, we revealed specific changes of the oocyte structures related to the condition. Analyzing meiotic spindle parameters and actin structure in the oocytes, we found distinct effects of COIA on the meiotic spindle size and actin cap formation. The spindles were larger and wider and with disorganized spindle fibers, and their poles were broader than in the control spindles. The actin cytoskeleton was affected too—instead of a normal actin cap anchoring the meiotic spindle to the oocyte cortex, a large and relatively thick peripheral ring was observed in the majority of CIOA oocytes. These defects, found in both M I and M II CIOA oocytes, suggest that osteoarthritis can affect the reorganization of tubulin and actin cytoskeletal structures during oocyte growth and meiotic maturation.

### 4.1. Meiotic Spindle Abnormalities and Chromosome Alignment

As seen in Table 1, almost all ovulated CIOA oocytes possessed a meiotic spindle (M I or M II). Despite their larger size and specific peculiarities, the spindles had a relatively normal shape. This suggests that spindle formation, even though affected, is nevertheless completed successfully during oocyte maturation under osteoarthritis influence. However, systemic inflammation factors could alter spindle morphogenesis in CIOA mouse ovaria.

The unique process of microtubule assembly in the absence of centrioles during meiotic spindle formation in the mouse combines two main mechanisms of microtubule nucleation: initial, dynein-mediated reorganization and formation of microtubules from acentriolar microtubule-organizing centers (MTOCs) at GVBD (germinal vesicle breakdown) stage, followed by (and combined with) chromatin-based kinesin-mediated MTOC reorganization at the beginning of meiotic prometaphase I [22, 23]. These mechanisms require activation of different pericentriolar material and centrosomal proteins involved in the nucleation of multiple microtubules from MTOCs and their later clustering. There are also other microtubule nucleation pathways such as chromatin-mediated microtubule nucleation and meiotic spindle assembly using Ran (Ras-like nuclear) GTPase, as well as the evolutionary conservative chromosomal passenger complex pathway based on the activity of Aurora B/C kinases associated with the kinetochores. These pathways are more important in oocytes of other species where MTOCs do not play the role they have in mouse oocytes [24]. Antiparallel microtubules of the overlap region are a region of binding of motor proteins transferring acentriolar MTOCs to the spindle poles. The action of kinases such as Aurora A kinase, polo-like kinases, and cyclin-dependent kinase 1 contributes to the spatial reorganization of MTOCs to focus the poles of the meiotic spindle [24–26].

Despite some specific abnormalities of CIOA oocyte meiotic spindles predominating in the group (longer, wider, with broad poles and disorganized microtubules), the majority of them had well-aligned chromosomes at the spindle equator of both metaphases I and II. The good metaphase plates in both CIOA and control oocytes suggest that spindle microtubules are attached successfully to kinetochores. It seems that the chromosomes in CIOA oocytes were aligned before or regardless of the action of osteoarthritis-related inflammatory factors. However, changes in the ovarian environment during meiotic maturation could cause imperfections of CIOA spindles as enlarged size or broader poles.

The proper attachment to and alignment of chromosomes on the spindle are controlled by the spindle assembly checkpoint (SAC) of the cell division. It recognizes unaligned microtubules and unattached chromosomes and prevents their segregation by delaying the onset of anaphase. Compared to mitosis, SAC of oocyte meiosis is less accurate and reliable, allowing meiosis progression through metaphase I arrest. This makes meiotic segregation of chromosomes and chromatids error-prone. Some authors associate the inefficient SAC control with the large ooplasmic volume [27]. Others ascribe it to the specific kinetochore orientation during meiosis I: at metaphase I, kinetochores are side-by-side oriented and attach both chromatids of the chromosome to the same spindle pole so that bivalents are bioriented and can be aligned at the spindle equator by the tension of kinetochore microtubules [28]. Abnormal kinetochore orientation or attachment can result in SAC activated arrest of meiosis or segregation error. Our results showing good quality and proper alignment of chromosomes in CIOA oocytes suggest reliable contact of spindle microtubules with chromosomes during earlier meiotic phases and movement toward the spindle equator. Nevertheless, one of the main differences between CIOA and control oocytes was the higher proportion of M I stage CIOA oocytes compared to the controls, as shown in Table 1. This lower maturation rate suggested metaphase I retention or arrest mediated by the spindle assembly checkpoint. Based on the observed spindle peculiarities of CIOA oocytes, we supposed that the inflammatory effect of osteoarthritis interfered with the complex process of meiotic spindle assembly, causing spindle enlargement, fiber disorganization, and/or SAC activation.

The effect of inflammation on oogenesis is complex and still poorly understood. According to a number of recent reports, folliculogenesis and ovulation share some molecular mechanisms with low-grade inflammation. The follicular fluid contains cytokines, some of them being proinflammatory, such as follicular TNF-*α*, IL-1, and IL-6, which in elevated levels affects negatively the oocyte quality. The level of IL-1 has been shown to depend on FSH follicular levels [29]. In the preovulatory follicle, prostaglandin, cytokine and chemokine secretion, and leucocyte infiltration are observed as a response to the luteinizing hormone surge causing ovulation. These findings support the “exotic” hypothesis of ovulation as an inflammatory process ([30], reviewed and reconsidered by [31]). At the same time, however, ovary-associated and systemic inflammatory conditions reduce female fertility, which is partly due to their negative effects on oogenesis [19]. In the context of our experimental work, control and CIOA animals were subjected to the same regimen of ovarian stimulation, animal housing, and cell processing, excluding any influence of these factors on the results. Hence, a low-level systemic inflammatory influence of osteoarthritis on oocyte maturation is the most probable cause for the differences between CIOA and control oocytes in our study.

The impact of osteoarthritis on oogenesis observed by us can be explained in the context of systemic inflammatory changes associated with this condition in humans [11] and in the same mouse model [14]. The particular factor(s) causing the observed reduced maturation rate of CIOA oocytes and morphological changes in their spindles could not be identified at this stage. The proinflammatory cytokine IL-6, characterized by elevated serum levels in osteoarthritis patients [32], causes deterioration of the meiotic spindle when applied to mature mouse oocytes [33]; however, the effects were not as specific as those observed by us, and the experimental setting was quite different.

Other teams have obtained meiotic spindle enlargement by treatments focused on particular molecules participating in spindle assembly and regulation. Injection of anti-Ran antibodies into immature mouse oocytes leads to the formation of large spindles, often with broad poles [34]. Uncontrolled activity of the motor protein Eg5 (kinesin-5) leads to spindle elongation in both mitosis [35] and oocyte meiosis [36]. Taxol treatment, which stabilizes microtubules and can induce their assembly, leads to enlarged spindles and nucleation of numerous microtubules in mouse oocytes [34, 37]. The latter result is particularly interesting in light of recent data that microtubule nucleation is the most important factor determining spindle size, especially in large cells [38], and the observation of astral microtubules in a number of CIOA oocytes in our present study. Normal mammalian oocyte spindles are barrel-shaped, with flat poles and without astral microtubules as a result of the absence of centrosomes [24]. Our enlarged CIOA spindles with disorganized microtubules, including astral microtubules, could indicate abnormally upregulated microtubule nucleation both around the poles and along the existing spindle fibers.

Another phenomenon that could be relevant is the mechanism of meiotic spindle assembly, described by some authors as the coalescence of short tiled microtubules into a bipolar structure in contrast to the long microtubules extending from each pole to the midzone of the mitotic spindle [39]. Such a discontinuous organization of the meiotic spindle fibers under the influence of unidentified inflammation-related disorganizing factor(s) could contribute to the “fuzzy” look of our CIOA spindles.

Whatever the precise molecular mechanisms, it seems that osteoarthritis inflammation factors can affect the ovarian environment of maturing mouse oocytes and influence the nucleation and assembly of spindle microtubules resulting in some imperfection of their arrangement along the spindle and its poles. Because the spindle peculiarities showed no specific differences between M I and M II, such a systemic effect of osteoarthritis could be presumed to act mostly on oocyte maturation before ovulation rather than on mature ovulated oocytes.

### 4.2. Fibrillar Actin Peculiarities

In our study, a specific defect of actin reorganization was observed in CIOA oocytes: the actin cap was replaced by a large, broad ooplasmic ring and the asymmetry of fibrillar actin distribution was lost. The essential asymmetry of oocyte meiosis is based on a specific reorganization of the subcortical actin cytoskeleton: microfilament nucleation forms an actin mesh, and a Ran-GTP-dependent, chromatin-derived spatial signal directs the spindle to the cortex. This is accompanied by peripheral actin accumulation as an actin cap that anchors the spindle. At the end of meiosis I, the extrusion of the first polar body is mediated by a contractile actomyosin ring [20, 40]. An intact actin cap is required also during meiosis II and in the ovulated oocyte arrested at metaphase II, because its anchoring of the M II spindle to the oocyte periphery is a prerequisite for successful completion of meiosis and fertilization [20].

A number of published reports describe disturbances of the actin cytoskeleton in mouse oocytes, caused by various factors. Depletion of certain actin-binding proteins, notably tropomodulin, decreases the ooplasmic actin mesh and cortical actin and impairs spindle migration and asymmetric division [41]. Depletion of kinesin motor protein KIF2A prevents spindle migration and actin cap formation, causing M I arrest of mouse oocytes because of spindle assembly checkpoint activation and, respectively, nonextrusion of 1st polar body [37]. Such a mechanism of disorganization could be related to the higher proportion of M I CIOA oocytes reported in our study, but the effect of KIF2A depletion reported by Chen and coauthors was accompanied by chromosome misalignment. By contrast, our CIOA oocytes had their chromosomes aligned even in large spindles with disorganized fibers. Moreover, the actin cytoskeleton formed a distinctive broad ooplasmic ring with no polarity in both M I and M II CIOA oocytes. A study on aged mouse oocytes [42] has found a reduction of the actin-driven oocyte polarity and lack of actin cap, accompanied by improper cortical granule distribution and a risk of polyspermy during fertilization. In our study, the same fixation protocol was followed for both CIOA and control oocytes, immediately after their isolation from the oviducts and removal of corona cells, so we can exclude postovulatory oocyte aging. The precise mechanisms generating the observed defects could not be identified within the current study, but it can be presumed that they reflected the impact of osteoarthritis on oocyte maturation in the ovaria of experimental animals. The defect of the actin distribution in CIOA oocytes looked quite specific for these cells and considerably more drastic than the spindle defects in this group. This abnormal microfilament organization compromises the quality of CIOA oocytes and, most probably, their ability to produce viable embryos.

## 5. Conclusion

Collagenase-induced osteoarthritis affects the cytoskeleton of ovulated mouse oocytes: (1) meiotic spindles of both M I and M II cells are longer and wider, their poles are broader, and disorganized fibers around the spindle and in its poles make it look fuzzy; (2) the actin cap is replaced by an ooplasmic ring showing no asymmetric distribution; (3) even abnormal, meiotic spindles were successfully formed in CIOA oocytes and allowed correct alignment of the chromosomes at the equator of both M I and M II. Further investigation is necessary to elucidate the precise molecular mechanisms of these CIOA-related cytoskeletal abnormalities observed in our study.

## Figures and Tables

**Table 1:** Absolute numbers and (in brackets) proportions of oocytes of each category.

	Number	Metaphase I	Metaphase II	Degenerated	Earlier stages
Control oocytes	209	86 (41.15%)	116 (55.50%)	2 (0.96%)	5 (2.39%)
CIOA oocytes	193	139 (72.02%)	51 (26.42%)	1 (0.52%)	2 (1.04%)
Total number	402	225	167	3	7

## Data Availability

The corresponding author will provide more details of the data upon request.
